# Low Intensity Ultrasound Promotes the Sensitivity of Rat Brain Glioma to Doxorubicin by Down-Regulating the Expressions of P-Glucoprotein and Multidrug Resistance Protein 1 *In Vitro* and *In Vivo*

**DOI:** 10.1371/journal.pone.0070685

**Published:** 2013-08-05

**Authors:** Zhen Zhang, Ke Xu, Yonghua Bi, Guibo Yu, Siwei Wang, Xun Qi, Hongshan Zhong

**Affiliations:** 1 Department of Ultrasound, China Medical University affiliated First Hospital, Shenyang, Liaoning, China; 2 Key Laboratory of diagnosis and interventional therapy of Liaoning Province, China Medical University affiliated First Hospital, Liaoning, China; 3 Department of Radiology, China Medical University affiliated First Hospital, Shenyang, Liaoning, China; University of Pecs Medical School, Hungary

## Abstract

The overall prognosis for malignant glioma is extremely poor, and treatment options are limited in part because of multidrug resistant proteins. Our previous findings suggest low intensity ultrasound (LIUS) can induce apoptosis of glioma cells. Given this finding, we were interested in determining if LIUS could help treat glioma by inhibiting multidrug resistant proteins, and if so, which pathways are involved. In this study, the toxicity sensitivity and multidrug resistance proteins of glioma induced by LIUS were investigated using CCK-8, immunohistochemistry, immunofluorency, and RT-PCR in tissue samples and cultured cells. LIUS inhibited increase of C6 cells in an intensity- and time-dependent manner. The toxicity sensitivity of C6 cells increased significantly after LIUS sonication (intensity of 142.0 mW/cm^2^) or Doxorubicin (DOX) at different concentration, particularly by the combination of LIUS sonication and DOX. The expressions of P-gp and MRP1 decreased significantly post-sonication at intensity of 142.0 mW/cm^2^ both *in vitro* and *in vivo*. The expressions of p110 delta (PI3K), NF-κB-p65, Akt/PKB, and p-Akt/PKB were downregulated by LIUS sonication and DOX treatment separately or in combination at the same parameters in rat glioma. These results indicate that LIUS could increase the toxicity sensitivity of glioma by down-regulating the expressions of P-gp and MRP1, which might be mediated by the PI3K/Akt/NF-κB pathway.

## Introduction

Brain glioma are malignant tumors which grow invasively and usually recrudescented after resection. The overall prognosis for malignant glioma is extremely poor, and treatment options are limited. Chemotherapy is one of the effective adjuvant therapies for tumors after surgery. But the results of previous studies show that chemotherapy does not significantly prolong the survival of patients with brain glioma because of multidrug resistance (MDR). Thus, it is important to find an effective method to overcome MDR in glioma. It is well known that ATP-binding cassette (ABC) transporter proteins, including P-glycoprotein (P-gp/ABCB1), multidrug resistance protein 1 (MRP1/ABCC1), and the breast cancer resistance protein (BCRP/ABCG2), induce MDR in tumors and actively extrude targeted therapeutic agents from the brain [1]–[3]. The antagonists of these proteins have been developed to overcome MDR, but many side effects were found during administration of these antagonists combined with chemotherapeutic agents. Developing an antagonist which causes less damage to normal brain tissue, while also improving the effectiveness of chemotherapy for brain glioma is an urgent clinical priority. Although doxorubicin is a routine chemotherapeutic agent in the clinical treatment of gliomas, glioma patients treated with Doxorubicin still may have issue with MDR, as the drug concentration in glioma cells does not achieve the therapeutic concentration; MDR proteins still exist in tissues and cells with Doxorubicin administration.

Ultrasound has been used as a safe diagnostic tool since the 1960’s. In recent years, high intensity focused ultrasound (HIFU) has been used to treat tumors [4], [5], and low intensity ultrasound (LIUS) is also used to treat chronic joint inflammation [6]. We have found that LIUS at specific parameters can induce apoptosis of glioma cells through the caspase-3, Bcl-2, and survivin signal pathways [7]. Some researchers have reported that ultrasound could decrease the expressions of P-gp [8] and MRP1 in liver cancer cells, which may be involved in the mechanism of multidrug resistant proteins. Taken together, these results indicate that LIUS could affect the activity of glioma cells. But whether the LIUS at similar parameters could induce cell proliferation is still unknown.

PI3K/Akt is an important signal pathway associated with cell proliferation and cell growth. This pathway consists of a catalytic subunit of p110 and modulated subunits of p65 and p85 and induces the expression of MDR proteins. PI3K induces PIP3 of second messengers on the cytoplasm membrane after activation, which leads to the activation of Akt. Akt is an important down-stream enzyme that modulates tumor cells in growth, survival, and apoptosis of tumor cells [9]. PI3K increases cell survival rates by Akt, inhibits pro-apoptosis signals, and activates anti-apoptosis genes [10], [11]. In stomach adenocarcinoma cells, LY294002, an inhibitor of PI3K, inhibits the activation of PI3K and reduces the activation of Akt and the expression of P-gp [12] **.** Akt may activate NF-κB through phosphoration, and then make NF-κB released from the cytoplasm to nucleus [13]. The NF-κB pathway may modulate the activity of gene *mdr1b* by the combined position in the upper-stream of gene *mdr*1b [14].

In this study, we investigate the efficacy of LIUS in the treatment of glioma, as well as potential mechanisms and pathways involved in the use of LIUS in the inhibition of multidrug resistant proteins. The effect of LIUS sonication on the proliferation of glioma cells and LIUS toxicity *in vitro*, as well as the expression levels of MDR proteins, P-gp and MRP1 *in vivo* and *in vitro* were investigated. We also examined potential LIUS influences on glioma cells via the signal pathway PI3K/Akt/NF-κB.

## Results

### Inhibitory Rate of C6 Cells Dependents on the Ultrasound Intensities and Duration Times *in vitro*

**Figure 1:**
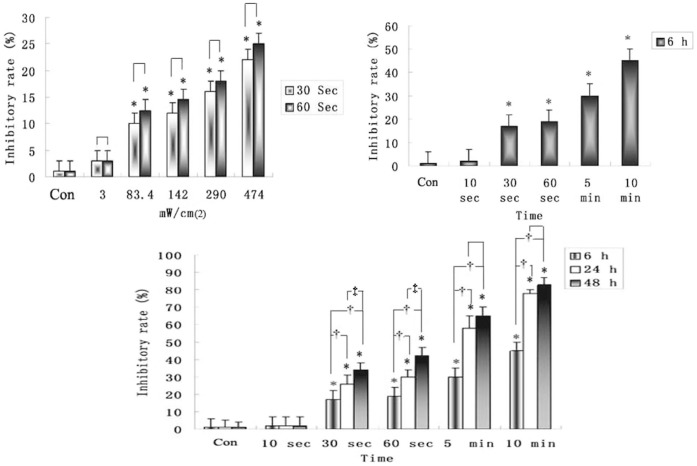
Inhibition rates of C6 cells measured by CCK-8 assay after LIUS sonication. A. Sonication durations are 30 s and 60 s, respectively. B. Durations are 10 s, 30 s, 60 s, 5 min, and 10 min, respectively at the same intensity (142.0 mW/cm^2^). C. The time points are 6, 24 and 48 h post-sonication at the same intensity (142.0 mW/cm^2^).*P<0.05 compared with control group. †*P*<0.05 compared with 6 h group. ‡ *P*<0.05 compared with 24 h group.

C6 cells were exposed to different intensities (3.0, 83.4, 142.0, 290.0 and 474.0 mW/cm^2^) of ultrasound for various durations (30 s and 60 s). The inhibition rates of C6 cells increased significantly with the increase in sonication intensities (*P*<0.05, ≥83.4 mW/cm^2^, Fig 1A). There was no difference between 30 s and 60 s at the same intensity (P>0.05). Therefore, in the following experiments, the 142.0 mW/cm^2^ intensity was used in all experiments.

At 142.0 mW/cm^2^, the inhibition rates of C6 cells increased significantly with the increase of sonication duration (10 s, 30 s, 60 s, 5 min and 10 min, *P*<0.05, Fig 1B) and at the time points from 6 h, 24 h to 48 h post-sonication (*P*<0.05, Fig 1C).

These results indicate that low intensity ultrasound inhibited the growth of C6 cells in an intensity- and time-dependent manner.

### Apoptosis and Membrane Permeability of Astrocyte and C6 Cells Induce by Different Ultrasound Intensities *in vitro*

The inhibition of C6 cells was observed post-sonication, indicating that ultrasound can inhibit the growth of tumor cells. To determine whether ultrasound of different intensities does harm to normal astrocyte cells post-sonication, primary astrocyte cells were used in TUNEL staining post-sonication.

**Figure 2:**
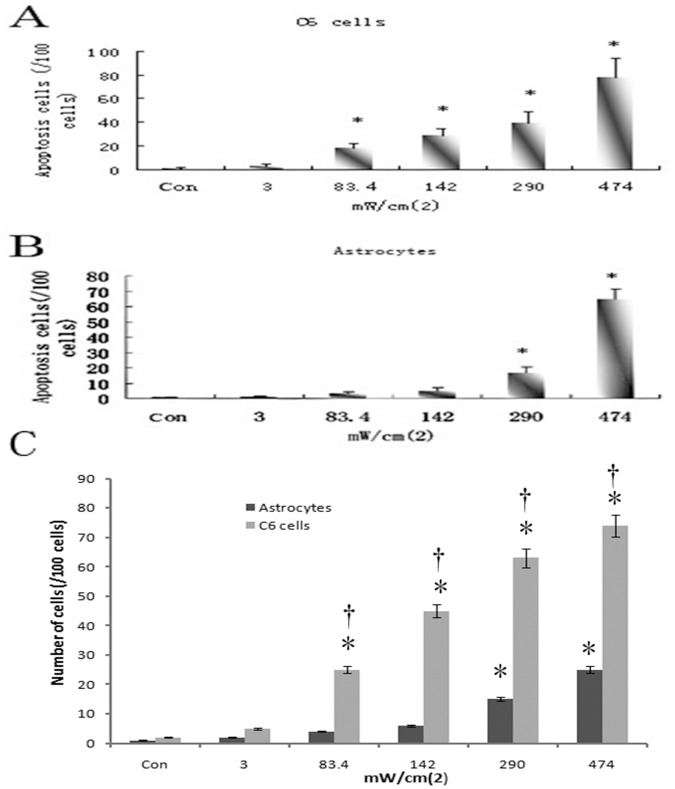
Apoptosis and membrane permeability of C6 cells and astrocytes are induced by LIUS sonication detected by TUNEL assay and propidium iodide (PI) staining. A. The number of apoptotic C6 cells. B. The number of apoptotic astrocytes. *P<0.05 compared with the control group (without sonication both in A and B). C. The number of astrocytes and C6 cells positively stained by PI staining. *P<0.05 compared with astrocytes.

Increasing numbers of apoptotic C6 cells were observed post-sonication at from lower to higher intensities compared with the control group without sonication (*P*<0.05, ≥83.4 mW/cm^2^, Fig 2A). Little apoptotic astrocyte cells were observed post-sonication at intensities of 3.0, 83.4, and 142.0 mW/cm^2^ (2±0.5/100 cells, 3±0.4/100 cells, and 4±0.3/100 cells, respectively) compared to the control group without sonication (2±0.3/100 cells). The number of apoptotic astrocyte cells significantly increased in groups of 290.0 and 474.0 mW/cm^2^ (15±2.5/100 cells and 65±14.5/100 cells, respectively) compared with the control group without sonication (*P*<0.05, Fig. 2B).

The cell number of C6 cells shown by PI staining increased 2 h after sonication at the intensities higher than 83.4 mW/cm^2^. The number of C6 cells increased significantly than that of astrocytes in groups of intensities higher than 83.4 mW/cm^2^ (Fig 2C, *P*<0.05). These results showed that LIUS could increase the permeability of C6 cells than that of astrocytes 2 h after sonication.

### LIUS Sonication and Doxorubicin (DOX) Increase C6 Cell Toxicity Synergistically *in vitro*

**Figure 3:**
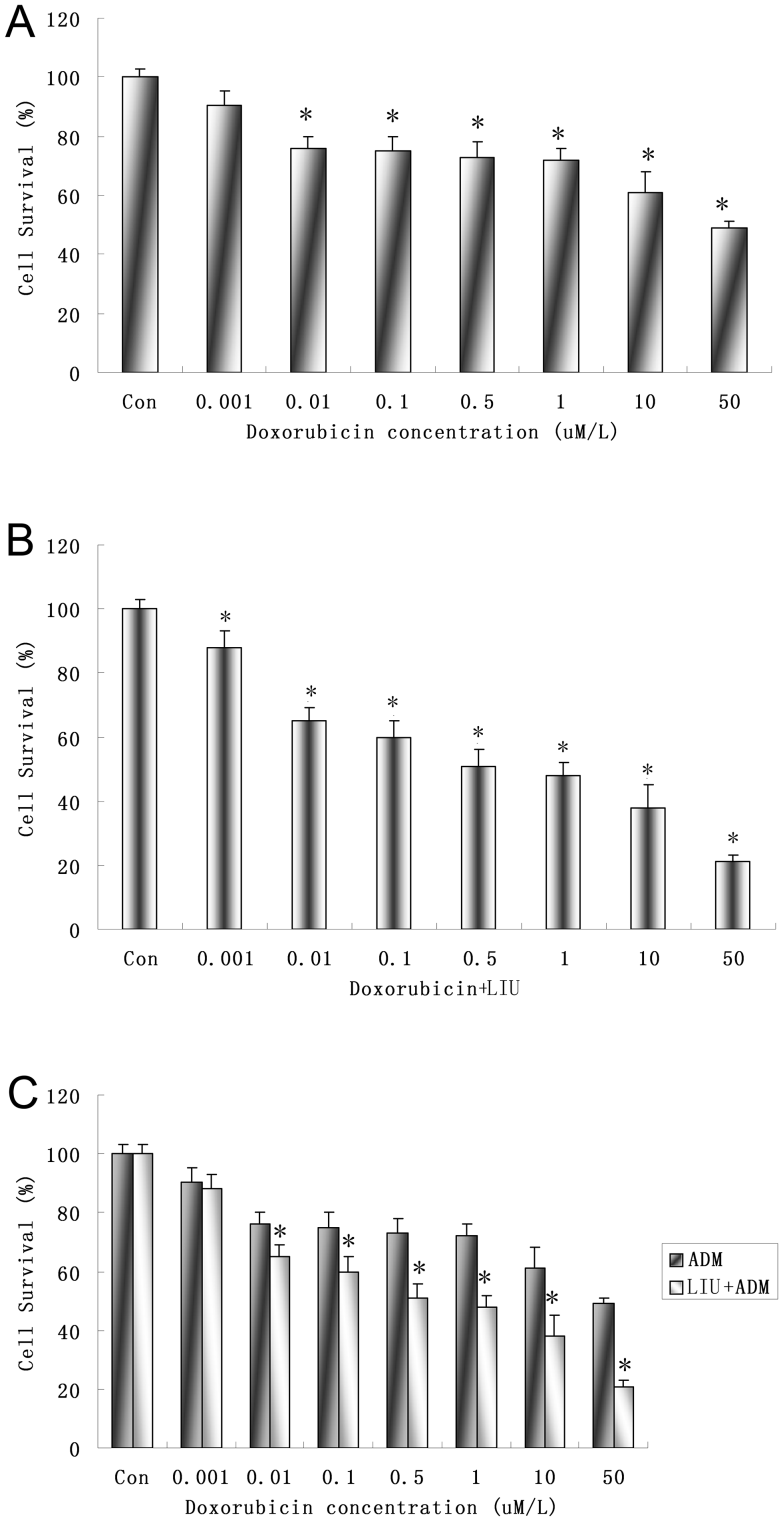
Inhibitory rates of C6 cells induced by Doxorubicin or/and LIUS sonication measured by CCK-8 assay. A. Treated by Doxorubicin (DOX) at different concentrations. *P<0.05compared with control group. B. Treatment at the combination of ultrasound (142 mW/cm^2^, 30 s) and Doxorubicin (DOX) at different concentrations. *P<0.01 compared with control group. C. Comparison of C6 cells inhibitory rates after treated by Doxorubicin (DOX) and the combination of sonication and DOX. *P<0.05 compared with DOX group at the same concentration.

C6 cells were treated by Doxorubicin (DOX) of different concentrations. The survival rates of C6 cells decreased significantly with increasing DOX concentration (≥0.01 µg/mL, *P*<0.05, Fig. 3A).

The combination of DOX of different concentrations and ultrasound (intensity of 142.0 mW/cm^2^ and duration of 30 s) significantly decreased the survival rate of C6 cells (*P*<0.05, Fig. 3B).

The survival rate of C6 cells exposed to DOX of different concentrations combined with LIUS sonication (intensity of 142.0 mW/cm^2^ and duration of 30 s) decreased significantly compared with those only exposed to DOX (*P*<0.05, Fig. 3C), suggesting that DOX and LIUS may increase the toxicity to tumor cells in a synergistic manner.

### Morphology Changes and Apoptosis Observed in C6 and Neural Cells of Rat Brain Glioma Model after LIUS Sonication *in vivo*

**Figure 4:**
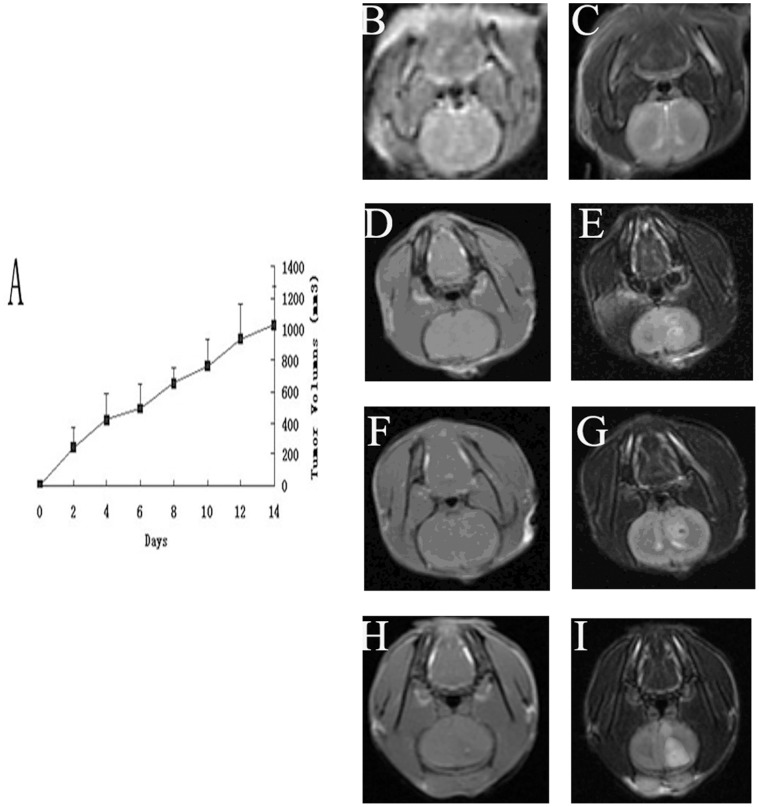
Volumes of brain glioma calculated by MRI scan. A Curve shows the volume of tumors on different days after transplantation. B, D, F, and H are images of T1 weighted signal. C, E, G, and I are images of T2 weighted signal. First day (B and C), 5 days (D and E), 10 days (F and G) and 14 days (H and I) after transplantation are shown.

MRI images were used to measure the tumor volumes within 14 days after glioma transplantation. We found that the volumes of glioma increased, and the curve was on the increase from the first day to the 14th day, especially 7 days after transplantation (Fig. 4 A). The glioma tumors in rats were shown in the magnet images of low signal in T1-weighted and high signal in T2-weighted (Fig. 4 B–I).

**Figure 5:**
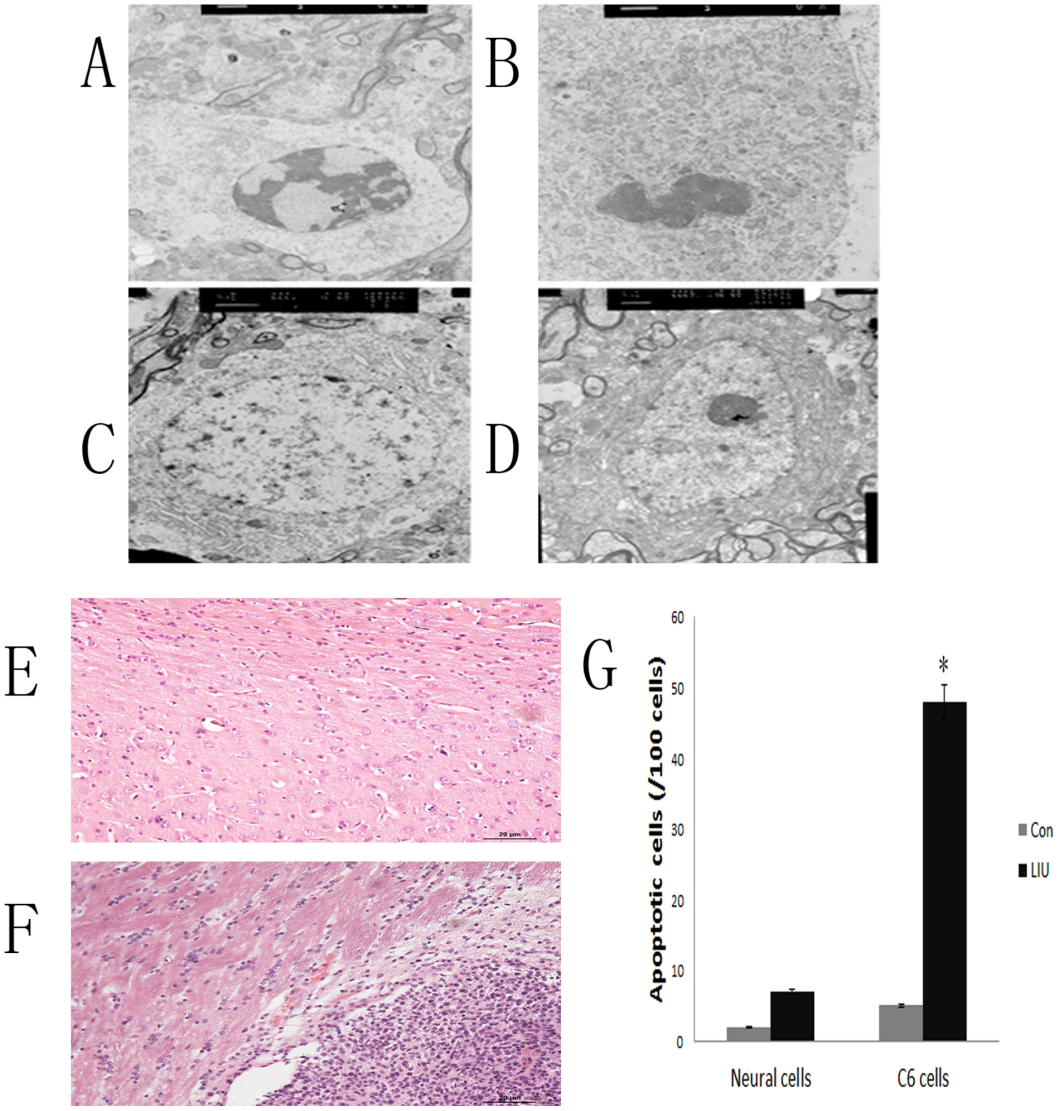
The changes in ultrastructures and apoptosis of C6 cells and astrocytes in rat brain observed by TEM (A–D), HE staining (E–F) and TUNEL assay after LIUS sonication. C6 cells of rat brain glioma are shown pre-sonication (A). Apoptotic body is observed post-sonication in C6 cells (B). Neural cell pre-sonication is shown (C). Neural cell membrane deformed and chromatin aggregation is observed post-sonication (D). Scale bars for ultrastructure are 200 nm. Normal rat brain tissues (E) and glioma tissues (F) 6 h post-sonication are shown without obvious destruction. Scale bar = 20 µm. *P<0.05 compared with the control group.

Apoptotic C6 cells were observed post-sonication by low intensity ultrasound. The C6 cells shrank and compacted. Condensation and segregation of the nuclear chromatin and the apoptotic body appeared. The neurons showed only membrane deformation and chromatin aggregation. No apoptotic neurons were observed (Fig. 5 A–D).

Under the same sonication parameters chosen *in vitro*, HE staining of rat brain glioma was performed. No obvious damage was observed under light microscope in both parts of normal brain and glioma area post-sonication. Only some red cells were observed in glioma and normal brain area post-sonication outside the macrovessel (Fig. 5 E–F).

Apoptosis of neural and C6 cells in rat brain glioma model were observed pre- and post-LIUS sonication. Under the parameters chosen in the study (intensity of 142.0 mW/cm^2^ and duration of 30 s), apoptotic tumor cells increased significantly post-sonication (*P*<0.05, Fig. 5 G), but apoptotic neural cells were not observed significantly (*P*>0.05, Fig. 5 G).

### Optimal Parameters for Ultrasound Sonication Chosen Through *in vitro* and *in vivo* Experiment

Based on the above findings, we concluded that an intensity of 142.0 mW/cm^2^ and duration of 30 s at probe frequency of 2 MHz should be chosen as the optimal parameters for both *in vitro* and *in vivo* experiments in this study, since the sonication inhibited the growth of C6 cells in an intensity- and time-dependant manner and induced apoptosis of C6 cells without obvious harm to neural and smooth muscle cells.

### LIUS Down-regulates Expressions of Multidrug Resistance Proteins P-gp and MRP1 *in vitro* and *in vivo* Post-sonication

**Figure 6:**
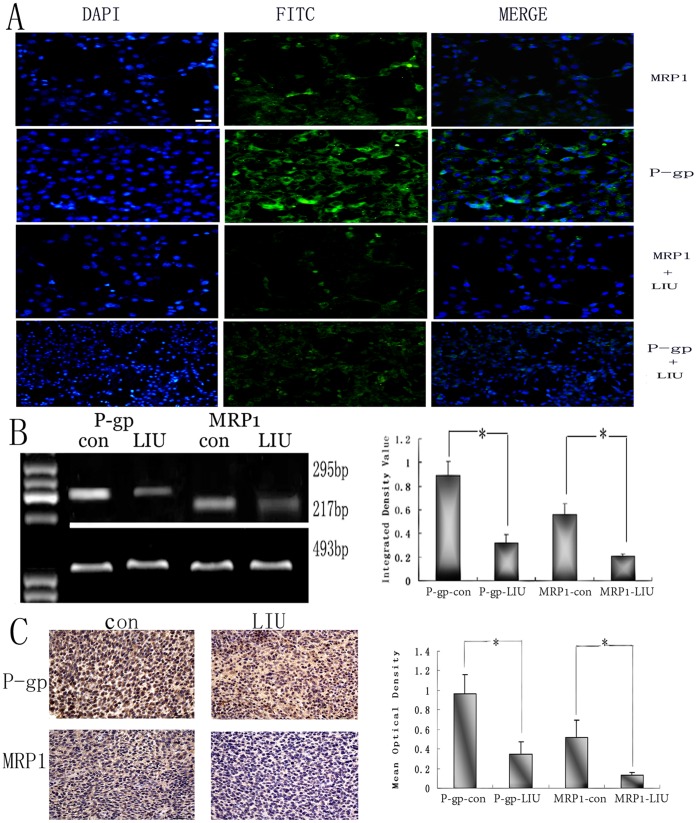
Expressions of P-gp and MRP1 after LIUS sonication. Down-regulation of P-gp and MRP1 proteins of C6 cells in vitro post-sonication (P-gp and MRP1) and pre-sonication (P-gp-con and MRP1-con) are shown by immunofluorency assay (A). Scale bar = 10 nm. The bands show that the expressions of P-gp and MRP1 of rat brain glioma at mRNA level reducing significantly after LIUS sonication. The analysis of bands shows integrated density value of P-gp and MRP1 post-sonication at mRNA level by RT-PCR assay (B). *P<0.05 compared with the control group. The expressions of protein levels of P-gp and MRP1 of rat brain glioma are shown by immunohistochemistry pre- and post- LIUS sonication. The expressions of P-gp and MRP1 reduce after LIUS sonication. The analysis of bands of mean optical density shows a significant down-regulation after LIUS sonication (C). *P<0.05 compared with the control group. Scale bar = 20 nm.

Under the conditions of probe frequency of 2 MHz, intensity of 142.0 mW/cm^2^, and duration of 30 s, the expressions of P-gp and MRP1 in C6 cells were observed pre- and post-sonication *in vitro*. Before sonication, both P-gp and MRP1 were highly expressed in C6 cells. However, 6 h post-sonication, the expressions of P-gp and MRP1 decreased significantly in C6 cells, observed as decreased fluorescence in the experimental group at post-sonication than in the control group at pre-sonication (Fig. 6 A).

RT-PCR results showed that mRNA expressions of P-gp and MRP1 reduced significantly *in vivo* post-sonication (Fig. 6 B), indicating that ultrasound could down-regulate the expressions of P-gp and MRP1 at the mRNA level.

The results of immunohistochemical staining *in vivo* were similar to those shown by RT-PCR belts (Fig. 6 C). The expressions of P-gp and MRP1 were down-regulated in experimental group compared with the control group. These results suggest that ultrasound can inhibit the expression of P-gp and MRP1 at the protein level.

### LIUS Down-regulates Expressions of Multidrug Resistance Proteins with DOX in a Synergistic Manner Related to the PI3K/Akt/NF-κB Signal Pathways in a Rat Brain Glioma Post-sonication

**Figure 7:**
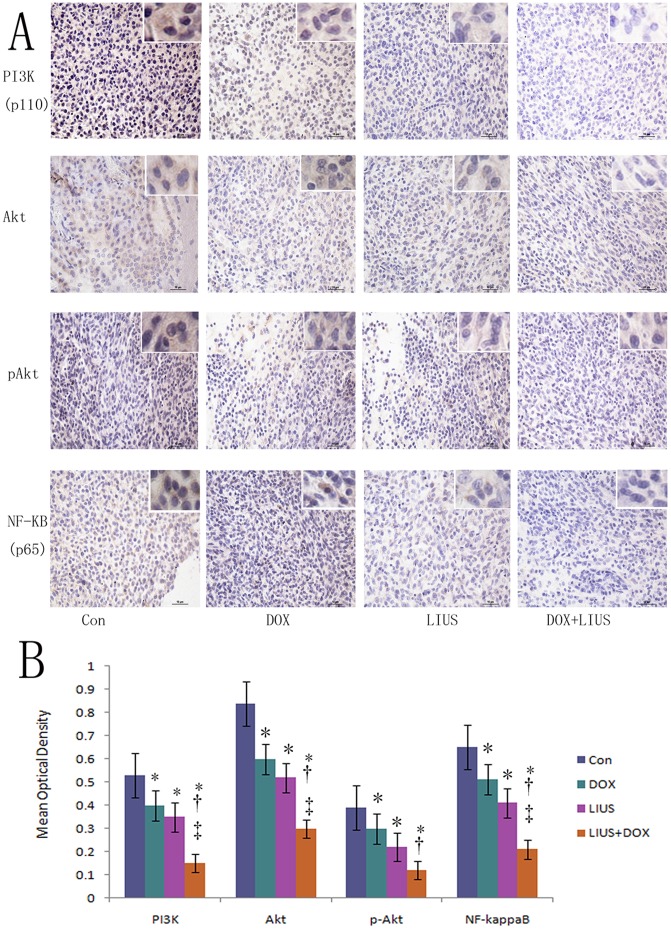
The influence on the expressions of PI3K\Akt\NF-κB signal pathways related proteins by DOX treatment and LIUS sonication separately or in combination shown by immunohistochemistry. PI3K-p110 delta, Akt, pAkt and NF-κB p65 signal proteins reduce significantly in rat brain glioma after LIUS sonication and DOX treated separately or in combination (A). B shows the analysis of the proteins by mean optical density. *P<0.05 compared with the control group. † *P*<0.05 compared with the DOX treated group. ‡ *P*<0.05 compared with the LIUS sonicated group. Scale bar = 10 nm.

PI3K, Akt and NF-κB are important signal proteins in the PI3K pathways. These pathways are related to the proliferation, survival, and apoptosis of brain glioma. The expressions of PI3K/Akt/NF-κB proteins were down-regulated significantly observed post-sonication and DOX treatment separately or in combination (*P*<0.05) by immunohistochemical staining. And the expressions of PI3K p110 delta, Akt, and NF-κB p65 reduced significantly in the combination of LIUS and DOX compared with LIUS or DOX groups alone (*P*<0.05, Fig. 7). These results showed a synergistic effect in the combination of LIUS and DOX.

## Discussion

Ultrasound reduced the proliferation of C6 cells and increased the toxicity of Doxorubicin to C6 cells at the probe frequency of 2 MHz in this study. A sonication duration greater than 30 s, and an intensity of greater than 83.4 mW/cm^2^ were found to be the most effective parameters *in vitro*. In order to induce less damage to normal cells, we determined the effect of ultrasound on C6 cells and glioma at the parameters of power of 142.0 mW/cm^2^ (>83.4 mW/cm^2^) and duration of 30 s at time point of 6 h post-sonication. The expressions of P-gp and MRP1 after LIUS sonication were decreased at the protein and mRNA levels *in vitro* and *in vivo*. Furthermore, in a rat glioma model, the expressions of PI3K/Akt/NF-κB proteins decreased significantly post-sonication, especially in the combination of LIUS and DOX.

In this study, we were able to demonstrate that the effect of ultrasound on the proliferation of tumor cells is associated with the intensity and the duration of ultrasound. Ultrasound is an effective tool that can influence the proliferation of tumor cells. The proliferation of C6 cells decreased significantly post-sonication, which is consistent with the results reported by Li et al [15]. The number of C6 cells decreased post-sonication at powers higher than 83.4 mW/cm^2^ and durations greater than 30 s. The number of tumor cells significantly decreased at 6, 24, and 48 h post-sonication. Therefore, the optimal parameters we chose in subsequent *in vitro* and *in vivo* experiments in this study were as follows: power of 142.0 mW/cm^2^, duration of 30 s, and time point of 6 h post-sonication.

Using light microscopy and HE staining, we found no obvious damage following sonication to either normal or glioma tissue. This suggests a mechanism of action for LIU at the protein or mRNA level. Our previous study suggests an effect on caspase-3, Bcl-2, and survivin, all proteins involved in apoptosis [7]. We found that the nuclear membrane in both tumor cells and normal cells was partly distorted under TEM in this study, which is consistent with the results of Guo et al [16]. The nucleus was damaged post-sonication in tumor cells but not in normal cells under the parameters used in the study, which is associated with cell proliferation or membrane permeability. Some important factors, such as NF-κB, have changed their locations in the nucleus and might be involved in the mechanisms of sonication.

We also found that ultrasound increased the toxicity of DOX to tumor cells in a synergistic effect after the combined application of ultrasound and DOX of different concentrations, which is in accordance with other reports [17], [18]. This result indicates that ultrasound could be used as an inhibitor for aiding tumor therapy. These effects of ultrasound could partly inhibit the effects of MDR proteins in tumor cells. Ultrasound may inhibit tumor growth by increasing the sensitivity of the tumor cells to chemotherapy drugs, as has been reported before [19]. It is well known that an advantage of ultrasound is that it is safer than other drug analogs which are more toxic to healthy cells. Ultrasound can selectively increase the toxicity of tumor cells, and, in our study, did no harm to the area without sonication. Future studies, using a rat glioma model, should investigate survival rates using combined application of low intensity ultrasound and DOX or other anti-cancer drugs.

No obvious damage was found in cells post-sonication at 142.0 mW/cm^2^, duration 30 s, 6 h post-sonication, with little changes in the structure of cell membrane in normal cells. This result indicates that ultrasound could induce non-thermal biological effects to break part of the cell structure temporarily, allowing for increased permeability of the cell membrane. These changes do no or little harm to normal cells under the parameters used in this study. We speculate that the intensity and duration of ultrasound might explain why ultrasound damaged tumor cells selectively. Different intensities and durations of ultrasound could have different effects on the tumor cells and/or normal cells. Tumor cells are more active in metabolism than normal cells. Under the parameters of ultrasound chosen in this study, the permeability of tumor cells increased significantly, and more and more DOX outside the cells moved into the cells to reach the necessary concentration for tumor cell apoptosis. Another possible reason is that the tumor area was targeted by ultrasound beams, one of the advantages of focused ultrasound.

Under the parameters we chose in this study, the down-regulating expressions of P-gp and MRP1 in tumor cells at the protein and mRNA levels were obvious in both *in vitro* and *in vivo* experiments post-sonication. These results strongly indicate that ultrasound sonication could reduce the expressions of P-gp and MRP1 in brain glioma cells, which are consistent with the results of liver tumor cells of Shao et al. [18]. Ultrasound affects the expression of MDR proteins in tumor cells through sonication, but the underlying mechanisms are still unknown. The apoptosis of tumor cells may play an important part. As we reported in a recently published article, the apoptosis of tumor cells is associated with the up-regulated expressions of caspase-3 and down-regulated expressions of Bcl-2 and survivin [7]. Other theories such as mechanical effects, cell proliferation, and surviving of molecules have also been put forward [20]–[26]. In this study, we investigated the molecular mechanisms involved of PI3K/Akt/NF-κB signal pathway.

The growth and proliferation of tumor cells were associated with PI3K/Akt/NF-κB signal pathway, which was found and affirmed in the *in vivo* experiment. PI3K/Akt was active in the cell proliferation, and NF-κB was active in the expression of gene *mdr1* [14], [27], [28]. The expression of the PI3K/Akt/NF-κB signal pathway was down-regulated post-sonication in rat glioma and after injection of DOX *in vivo*. And the combination of DOX and LIUS *in vitro* and *in vivo* could down-regulate the expressions of PI3K/Akt/NF-κB in a synergistic manner. The down-regulation of PI3K/Akt/NF-κB reduced the expression of P-gp and MRP1 in this study, which is consistent with the results of Hien et al., who reported suppression of MDR1 via NF-κB pathway in breast cancer cells [29]. This result supports the theory that low intensity sonication affects glioma at the molecular level.

In summary, we concluded that ultrasound at optimal parameters could improve the sensitivity and toxicity of tumor cells to DOX safely and selectively during chemotherapy, without damaging normal neural cells in rat glioma. The related MDR proteins, P-gp and MRP1, are down-regulated by ultrasound at the same parameters, in which the down-regulation of PI3K/Akt/NF-κB signal pathways may be involved. These results indicate that LIUS could increase the toxicity sensitivity of glioma by down-regulating the expressions of P-gp and MRP1, which might be mediated by the PI3K/Akt/NF-κB pathway. The exact mechanism of how PI3K/Akt/NF-κB signal pathway regulates the expressions of P-gp and MRP1 in glioma by sonication needs further investigation.

## Materials and Methods

### Culture of C6 Cells

C6 cells kindly provided by the Department of Neurosurgery, the First Hospital, China Medical University were purchased from a typical cell culture collection Committee of the Chinese Academy of Sciences Library. The 10% serum medium consisted of Dulbecco’s modification of Eagle’s Medium (DMEM) plus 10% fetal bovine serum (FBS, Hyclone, Thermo Fisher Scientific Inc., USA). All cells were incubated at 37°C in a humidified atmosphere containing 5% CO_2_. The cells were cultured and then harvested for *in vitro* and *in vivo* experiments.

### Culture of Astrocyte Cell

Astrocyte cells were used as a normal cell control compared with tumor cells in *in vitro* experiment in TUNEL assay. The astrocytes used in this study were obtained from the rat brain, and operated under the permission of the Animal Ethics Committee of China Medical University. The protocols for animal use and care were approved by the Institutional Animal Care and Use Committee of China Medical University. Cerebral astrocytes were obtained from brain cortices of 2-day-old rats. Meninges, large vessels and white matter were removed carefully and grey matter pieces were dissociated mechanically into small pieces in ice-cold D-Hanks. The cortical pieces were disaggregated in trypsin (2.5 mg/ml) diluted with Ca^2+^/Mg^2+^ free PBS at 37°C for 20 min. After centrifuged at 150 (×g) for 5 min, the precipitate was re-suspended in the medium with 10% FBS. The suspension was filtered through a 10-mm-pore-size nylon mesh and washed by medium with 10% FBS. Finally, the filtrate was centrifuged at 150 (×g) for 5 min and then re-suspended with culture medium containing 10% FBS, penicillin (100.0 U/ml), streptomycin (100.0 mg/ml) and plated on 25 cm^2^ plastic dishes pre-coated with poly-L-lysine (0.1 mg/ml) at 37°C and 5% CO_2_. On the second day, it was changed with new medium. Then the culture medium was changed every 2 days. When the confluence reached 80% (the 4–5th day), the plastic dishes was shaken at 220 rpm for 18 h at 37°C to purify the astrocytes. The purified astrocytes were passaged by a brief treatment with trypsin (2.5 mg/ml)-EDTA (0.2 mg/ml) solution and the second passage was used to the following experiment.

### Parameters of the Ultrasound *in vitro* and Grouping

An ultrasound beam of 2 MHz was generated by a pulse-waved transducer from an EMS-9 ultrasound apparatus (Shenzhen Delica Company, China) and sonicated vertically upward from the bottom of a polystyrene 96-well plate through a water tank in the *in vitro* experiment [7]. Each sample was sonicated separately by moving the plate to the target of the focused ultrasound. A coupling agent was used between probe and culture plate as described previously [7]. In this study, we carefully created several ultrasound fields to almost completely eliminate standing wave effects.

The same parameters of the transducer in the *in vitro* experiments in each group were set as follows: repetition frequency of 400 Hz, diameter of 1.56 cm (area of 2.0 cm^2^), burst length of 0.25 ms, focusing distance of 5 mm, focusing length of 10 mm, duty cycle of 10%. The optimum parameters were chosen by *in vitro* cell experiment; these same parameters were used for the *in vivo* experiment.

First, we employed varying sonication intensities in the *in vitro* experiment. Groups (duration of 30 s and 60 s) were set according to acoustic powers of 3.0, 83.4, 142.0, 290.0, and 474.0 mW/cm^2^, with the corresponding TI of 0.05, 1.36, 2.32, 4.74, and 7.75. Then, the optimum duration was chosen from 10 s to 10 min. Groups were set according to the sonication durations as 10 s, 30 s, 60 s, 5 min, and 10 min, respectively. At last, the optimum time point post-sonication to obtain the results was observed. The time points were set as 6, 24, and 48 h at the optimum intensity and different duration times chosen from above post-sonication. The ultrasound contrast Optison™ (GE Healthcare, Milwaukee, WI, USA) at 10% (v/v) was added to the cells in each group (both in experimental and control groups) 10 s before each sonication.

### Animal Model and Grouping

Adult female Wistar rats (180 to 200 g, n = 32) were provided by the Experimental Animal Center, China Medical University. All animal experiments were carried out in accordance with the National Institute of Health Guide for the Care and Use of Laboratory Animals and the Society for Neuroscience Guidelines for the Use of Animals in Neuroscience Research. The rat model of C6 glioma was established as follows: C6 glioma cells (provided by the Department of Neurobiology, China Medical University) were cultured in DMEM (Sigma-Aldrich, USA) with 10% fetal calf serum (Gibco, USA) at 37°C and 5% CO_2_, and harvested at log phase by centrifugation. Rats were anesthetized with 10% chloral hydrate (0.3 mL/100 g, intraperitoneal injection). Ultrasound window was created by craniotomy, with a piece of 3 mm ×3 mm skull removed from 1 mm anterior to sutura coronaria and 3 mm lateral to sagittal suture on the right. The suspension of C6 glioma cell was injected by a Hamilton syringe, under the direction of stereotaxic apparatus [30]. The coordinates were 5 mm lateral to the bregma and 4.5 mm deep to the head of caudate nucleus in the right brain. The skin was then sutured. The rats were exposed to ultrasound (with Optison) 14 days after the injection of C6 glioma cells. Within 14 days pre-sonication, the volume of the tumor was observed by MRI.

The tumor-bearing rats were divided into four groups and received LIUS sonication for 30 s (LIUS group), vein injection at a doxorubicin dose of 5 mg/kg via the tail vein (DOX group), LIUS and DOX at the same parameters (DOX+LIUS group) and no treatment (control group) (*n = *8 each group) 14 days after the operation, respectively. The rats in the experimental groups were killed at 6 h post-sonication and/or DOX injection. The ultrasound beam was targeted through the boneless window of rat right hemisphere by stereotaxic apparatus. The parameters of the transducer sonication in the experimental group were set as follows: repetition frequency of 400 Hz, sonication time of 30 s, diameter of 1.56 cm (area of 2 cm^2^), focusing distance of 5 mm, focusing width of 20 mm, burst length of 0.25 ms, radius of curvature of 5 mm, acoustic power of 142.0 mW/cm^2^, with the corresponding TI of 2.32. The ultrasound contrast Optison™ (GE Healthcare, USA) was injected intravenously (50 µL/kg) through the femoral vein 10 s before each sonication.

### Cell Proliferation Assay *in vitro*

A total of 100 µL suspension of C6 cells was put into a 96-well plate and incubated at 37°C for 48 h post-sonication at different intensities and duration of sonication *in vitro*. 10 µL CCK-8 was added into the plate and incubated for 4 h. The absorption rate at 450 nm was measured, and the reference wavelength was at 600 nm. The inhibitory rate (IR) was calculated according to the following equation: IR (%) = (1-As).Ac^−1^×100%, in which As denotes the absorption rate in experimental holes (including C6 cells, DMEM, CCK-8 post-sonication) and Ac denotes the absorption rate in control holes (including C6 cells, DMEM, CCK-8 without sonication).

### Sensitivity of Doxorubicin

CCK-8 assay was performed to assess the chemo-sensitivity of C6 cells to anticancer drugs. In brief, 6 h after cultivation, the medium was removed and replaced with fresh medium containing different concentrations (0, 0.001, 0.01, 0.1, 0.5, 1, 10, and 50 µg/mL, respectively) of DOX, an anti-cancer drug, and incubated for 48 h. Groups were divided according to the concentrations of DOX. Relative inhibitory rate (IR) of C6 cells was calculated according to the following equation: IR (%) = (As-Ab) (Ac-Ab)^-1^×100%, in which Ab denotes medium added with CCK-8 but without C6 cells and LIUS sonication.

### Sensitivity of Doxorubicin and Ultrasound

As described above, after incubation with DOX at different concentrations (0, 0.001, 0.01, 0.1, 0.5, 1, 10, and 50 µg/mL, respectively) for 48 h, C6 cells were sonicated for 30 s, and then incubated for 6 h post-sonication. Groups were divided according to the concentrations of DOX, combined with the LIUS sonication at the same parameters. CCK-8 was added into the suspension and incubated for 4 h before measurement. Relative inhibitory rate of C6 cells was calculated according to the formula listed above.

### Hematoxylin and Eosin (HE) Staining *in vivo*

The samples of rat gliomas were cut into slices. The slices in the control (without sonication) and experimental groups exposed to sonication were fixed with 4% multiple formaldehyde for 5 min, stained with hematoxylin for 5 min and eosin for 2 min before dehydration. Photographs were taken with an Olympus BX 60 Upright microscope (Olympus Company, Tokyo, Japan).

### Terminal Deoxynucleotidyl Transferase-mediated dUTP Nick End Labeling (TUNEL) Assay *in vitro* and *in vivo*

An in situ Cell Death Detection kit (Roche Applied Science, Cat # 11 684 817 910) was used in TUNEL assay. The cell slices of the rat astrocyte cells and C6 cells *in vitro* exposed to sonication at different powers (five experimental groups from 3.0 to 490 mW/cm^2^, and a control group) 6 h later were stained according to the manufacturer’s instructions. The tissue slices of the rat brains *in vivo* exposed to sonication for 30 s at 142.0 mW/cm^2^, both the normal and glioma tissue, were also stained according to the manufacturer’s instructions. Briefly, the cells or tissue slices were pretreated with proteinase K (Roche, Germany) for 30 min at 37°C and then exposed to TUNEL reaction mixture, which contains terminal deoxynucleotidyl transferase and nucleotides including fluorescein isothiocyanate-labeled dUTP (37°C). After 60 min, an anti-fluorescing peroxidase-linked antibody was added, followed by 30-min incubation at 37°C, and then the nuclei were stained. A subpopulation of apoptotic cells scattered throughout the cell panel was intensely stained (green) by TUNEL assay *in vitro*. Tissue slices were continuously stained brown, for the apoptotic cells. The number of apoptotic cells was counted and analyzed, and these cells were photographed with an Olympus BX 60 Upright Fluorescence microscope (Olympus Company, Tokyo, Japan) over 30 fields.

### Immunofluorency *in vitro*

Two hours after treatment at different intensities by sonication, astrocytes and C6 cells were treated with Propidium Iodide solution (PI, Sigma-Aldrich, Oakville, Ontario) at a concentration of 1.5 µmol/L to allow the identification of damaged cells with perforated membranes. They are then washed with PBS and fixed with 4% formaldehyde solution (Sigma-Aldrich, Oakville, Ontario). After another PBS washing, cell nuclei are stained with DAPI (Sigma-Aldrich, Oakville, Ontario) at 1 µmol/L. The positively stained cells were visualized and counted with an Olympus BX 60 Upright Fluorescence microscope.

Indirect immunofluorescence was used to detect the localization and expression of P-gp and MRP1 proteins (Sigma-Aldrich, Inc., USA) in C6 cells. Cell slides were incubated with the primary antibodies (1∶100 dilution) at 4°C overnight or at room temperature for 1 h. After being rinsed with PBS, the slides were incubated with FITC conjugated mouse anti-rabbit IgG (according to different primary antibodies, 1∶100 dilution, Sigma-Aldrich, Inc., USA) for 40 min at room temperature, shielded from light. The sections were covered by cover-slip and sealed by glycerin. Fluorescence was visualized and documented with an Olympus BX 60 Upright Fluorescence microscope.

### Immunohistochemistry *in vivo*

Crystal sections (10 µm) were mounted on glass slides coated with poly-L-lysine, dried, and stored at −25°C for histological and immunohistochemical assay. Quenching of endogenous peroxidase was performed by incubation for 30 min, and nonspecific binding was minimized by incubation with blocking solution for 20 min. The sections were counterstained with Mayer’s hematoxylin and eosin to confirm the presence of tumor tissue. The sections stained with SABC were incubated with polyclonal rabbit anti-P-gp, -MRP1 (1∶150 dilutions, Santa Cruz Biotechnology, Inc., USA) and polyclonal rabbit anti-PI3K p110 delta, anti-Akt, anti-pAkt (Ser473), and anti-NF-κB p65 antibodies (1∶150 dilutions, Santa Cruz Biotechnology, Inc., USA) at 4°C overnight. Negative controls were carried out using the same procedure with PBS substituting primary antibody. For semi-quantitative measurements of these proteins’ densities, the slides were photographed and analyzed. Images were visualized and processed with Chemi Imager 5500 V2.03 software (AlPha InnCh,USA) and quantified.

### MRI Imaging

MRI images acquisition was performed on a 3T MRI system (GE, USA). The rats were anesthetized with 10% chloral hydrate in a box for 5 min for induction, and then placed in a wrist joints holder, in which their heads and bodies were fixed to the cradle with tape and ear pins. The rats in the magnet were monitored visually online through a monitoring and gating system, and connected by fiber optics. The rats underwent MRI scanning at postimplantation days 0 to 14 to detect the tumor volume.

In vivo multi-slice images of rat brains were acquired in the horizontal plane (5 slices; slice thickness 1.5 mm; field of view = 42 mm × 32 mm) and in the coronal plane (5 slices; slice thickness 1.5 mm; field of view = 32 mm × 32 mm). The parameters of MRI images were set as follows: T1WI: TE 4.25; TR 150; FOV 80×80; matrix 256×256; NEX 1, 1.9 thk/0.1 sp. T2WI: TE 78; TR 6100; FOV 80×80; matrix 256×256; NEX 2, 1.9 thk/0.1 sp. The total imaging time for each animal was about 1 h. The tumor volume was measured with GE company ADW4.4 software.

### Transmission Electron Microscopy

Catheters were inserted into the aorta through the left cardiac ventricle following anesthesia. First, the rats’ hearts were infused by 100 mL or more of normal saline until the infusion fluid flew out clear, followed by a second infusion with 200 mL of 4% paraformldehyde. Implanted tumors were excised from the rat brains. The tumor tissues were randomly selected, diced (1 mm^3^), and fixed in 1% glutaraldehyde for 2 h at 4°C. After that, according to the standard procedures, the tissues were embedded, sectioned to semi-thin and ultra-thin slices, and stained with uranyl acetate and lead citrate. The microstructure of glioma cells was examined under transmission electron microscope (JEM-1200EX, Japan) at an accelerating voltage of 80 kV (*n = *8 each group).

### Reverse Transcriptase of Polymerase Chain Reaction (RT-PCR)

The rats (*n* = 8 each group) were sacrificed by decapitation immediately post-sonication. The whole glioma tumor was dissected and placed in cold (4°C) D-Hank’s solution without calcium and magnesium, and manually minced and subsequently homogenized in a glass homogenizer. The tissues were washed with cold D-Hank’s and centrifugated for 5 min at 4°C and 3000 *g*. Myelin was removed by re-suspending the pellet in cold DMEM containing 15% Dextran (Sigma-Aldrich, USA) at 4°C and centrifugating at 10,000 g for 15 min at 4°C. After centrifugation, the myelin was concentrated at the upper level of the medium and could be easily removed. The remaining pellet was washed twice in DMEM with a centrifugation at 3000 g for 5 min at 4°C. Then tumor samples were obtained. Total RNA was prepared from tumor using TRIzol reagent (Invitrogen, USA). cDNA synthesis, amplification, and detection of distinct P-gp and MRP1 were carried out. RT-PCR was performed according to the manufacturer’s protocol. A total of 30 PCR cycles were selected for P-gp, MRP1, and βeta-actin. βeta-actin served as a control in each experiment. Products were electrophoresed on a 2% agarose gel. Gel photos were taken using Chemi Imager 5500 gel image analysis instrument (AlPha InnCh, USA).

The forward and reverse primer sequences were 5′-GCATTCTGGTATGGGACTT and 3′-GTCTTTTCGAGACGGGTA for P-gp (ﬂanking a 295-bp region), 5′-CCCTGAAGAGCAGTGACCTC and 3′- TAGGCTTGGTGGGATCTTTG for MRP1 (ﬂanking a 217-bp region), and 5′- TCTGTGTGGATTGGTGGCTCTA and 3′-CTGCTTGCTGATCCACATCTG for βeta-actin (ﬂanking a 493-bp region).

### Statistical Analysis

All experiments were repeated at least three times. Data are expressed as mean ± SD and were analyzed with SPSS Release 11.5 (SPSS Inc., US). A Student’s *t* test was performed to determine significant differences between two groups. One-way ANOVA and *post hoc* comparisons (*Bonferroni* test) were used to determine significant differences among multiple groups. A *P* value of 0.05 or less was considered statistically significant.
